# From Registration to Publication

**DOI:** 10.1371/journal.pmed.0010046

**Published:** 2004-11-30

**Authors:** The PLoS Medicine Editors

## Abstract

The *PLoS Medicine* editors argue that all trial registries, reports, and databases should be freely available online

In a compelling essay in this issue of *PLoS Medicine*, Mike Clarke, the director of the United Kingdom Cochrane Centre, lays down a challenge to clinical researchers and journal editors [1]. He argues that researchers should do a study only if there is a systematic review that shows that the new study is needed. If no review exists, the researchers should do one themselves before embarking on their research. And journals, he argues, should publish a study only if an updated systematic review is incorporated into the study, or published alongside it or shortly thereafter. How should editors respond to his challenge?

First, we would argue that by the time a paper is sent to a journal, it is surely too late in the process to be insisting on systematic reviews. If a clinical trial report meets our criteria for originality, importance, and quality, it makes little sense for us to reject it just because the authors failed to systematically review the literature when designing their study. The time to mandate that researchers do a review is much earlier—when they apply for funding, register their trial, or seek ethics committee approval.

There is no doubt that the best research builds on previous knowledge. But unfortunately, the current medical publishing system hides much of this knowledge behind subscription or “pay per view” charges, which discriminates against researchers who do not work for well-funded institutions. A group of researchers in Indonesia, for example, recently told a depressingly familiar story of trying to search the medical literature in preparation for a research project [2]; access barriers got in their way. So our second response to Clarke's challenge is that it will remain difficult for researchers, particularly in resource-poor settings, to do systematic reviews unless the medical literature is made a freely available public resource.

Many clinical trials, especially negative ones, remain unpublished, which prevents researchers from reviewing all the data on an important health issue. There are two main reasons why certain trials are not published: one is that the pharmaceutical industry has a long history of suppressing data that are commercially unfavorable and the second is that medical journals and the popular media favor publication of positive over negative trials (after all, negative trials do not make for a provocative newspaper headline). While we support the recent announcement on trial registration by the International Committee of Medical Journal Editors—as a condition of considering a trial for publication, member journals will require registration of the trial in a public trials registry [3]—we believe that this policy addresses only part of the problem.

The scientific literature will remain biased unless the publishing industry changes its practices and provides a place where the results from all registered trials can be published. *PLoS Medicine* is committed to publishing high-quality negative trials. In this issue, for example, we publish an important randomized controlled trial of a malaria vaccine in 372 Gambian men, which found that the vaccine was ineffective at reducing the natural infection rate.

The internet makes it possible for every single clinical trial to be publicly and seamlessly tracked through three tiers. The first tier is registration in a publicly available database. The second is the publication of a peer-reviewed summary of every trial, regardless of its outcome, in a traditional journal format, with annotations and critiques that help readers understand the trial's implications. The third is the deposition of detailed trial data in a structured, computable format that allows sophisticated searching and analyses across trials. This format will allow the development of better tools to help clinicians apply trial results to their practice. For trial data to be as useful as possible, all three tiers must be publicly accessible. Assessment of each trial's validity is critical, but should not stop crucial information about all trials being placed in the public domain.

Trial registries exist, such as ClinicalTrials.gov and the International Standard Randomised Controlled Trial Number registry. Moreover, many trials are registered in a semi-public database maintained by the United States Food and Drug Administration, and there are compelling arguments (which Turner articulates in an essay published online ahead of our December issue [4]) for making this a truly public resource. Publicly accessible trial databases (such as the Trial Bank Project at http://rctbank.ucsf.edu) are under development. And as a publisher committed to open access, PLoS will provide the second, essential tier—journals capable of peer reviewing and publishing an annotated report of every trial. Traditional medical journals, with their subscription-based model, are unlikely to be able to provide this service, because in order to attract subscribers they need to publish only the highest-profile trials. We believe that an open-access model—in which the research funder pays a publication fee to recover the costs of peer review and for hosting the report on a secure server—is the best mechanism for creating such venues. We are working to make that happen.

Returning to Clarke's challenge, our final response is to say that we have a bold vision of a freely accessible online world of clinical trials—from registration to annotated summaries to trial databases. That world would be even richer if every systematic review were made freely available. We challenge the Cochrane Collaboration to put the full text of all of its reviews into the public domain. We hope the Cochrane Collaboration will join us in the open-access revolution.
